# Information scanning – keeping in touch with best practice in breastfeeding

**DOI:** 10.1186/1746-4358-1-15

**Published:** 2006-09-11

**Authors:** Ellen McIntyre

**Affiliations:** 1 Primary Health Care Research & Information Service (PHC RIS), Department of General Practice, Flinders University, GPO Box 2100, Adelaide SA 5001, Australia

## Abstract

This paper describes some of the methods practitioners can use to scan sources for up-to-date information about best practice in breastfeeding.

## Background

There is so much we still need to know about how best to enable mothers to successfully breastfeed. In addition, we are all time-poor in an ever increasing information-rich environment. This paper describes some of the methods practitioners (those directly involved with helping mothers) can use to scan the environment for up-to-date information about best practice in breastfeeding. By keeping in touch – with other practitioners and International Board Certified Lactation Consultants (IBCLC), with researchers, with decision makers, and with research findings – practitioners can ensure they help mothers most effectively.

## Keep in touch with other practitioners, especially IBCLCs

Attend relevant conferences, seminars and workshops in your area but also once in a while go to a conference in another state or region or even overseas. While there is an added cost, it is worthwhile broadening your horizons beyond your local area. While at conferences, make the most of the opportunity and network with other delegates. To keep informed about conferences, join your local professional lactation organization and the International Lactation Consultants Association (ILCA) [1]. Check their websites as well as the International Board of Lactation Consultants Examiners (IBLCE) website regularly to keep up to date with issues pertinent to this profession [2]. Their links are also worth viewing. You might also organize to visit other IBCLCs at their workplaces.

Lactnet, an email list of health professionals and others interested in discussing issues of breastfeeding contains a wealth of information [3]. Although it can be very time consuming to read the emails submitted by those among the 3300 plus subscribers, it does have an archival facility to assist with searching specific topics.

## Keep in touch with researchers

As mentioned above, attend conferences but also make the effort to talk to the researchers who are presenting their work. If you have read some of their work, send them an email (most papers list author contact details) if you wish to know more or wish to share your ideas. Who knows, it may lead to a collaborative research partnership to conduct research to answer your questions.

## Keep in touch with relevant decision makers, in particular government agencies

The health promotion section of many state or regional Health Departments are doing something about breastfeeding. Why not contact them to find out what is/is not happening about breastfeeding in your area? They may be interested in your ideas and support. This happened in South Australia, where several IBCLCs were invited to become part of the State Breastfeeding Reference Group which developed a breastfeeding action plan resulting in the implementing of several breastfeeding initiatives.

## Keep in touch with research findings

PubMed is a free access website to research findings [4]. The Cochrane library which provides evidence to inform healthcare decision-making, is available free in many countries eg Australia, New Zealand, Scandinavia, UK [5].

Many breastfeeding organisations also publish research findings and promote breastfeeding initiatives on their websites, including:

Australian Breastfeeding Association (ABA) [6]

Breastfeeding Friendly Hospital Initiative, Australia [7]

UNICEF Baby Friendly Initiative [8]

International Baby Food Action Network (IBFAN) [9]

LACTMED – Drugs and Lactation Database [10]

United Nations International Children's Emergency Fund (UNICEF) [11]

World Alliance of Breastfeeding Action (WABA) [12]

World Health Organisation (WHO) [13]

La Leche League International (LLLI) [14]

In some cases you can subscribe to email alerts and receive regular emails advising you of new additions to their website. PubCrawler, a free "alerting" service that scans daily updates to the PubMed databases makes it easy to keep up with the latest on a particular issue [15].

Subscribing to the Australian Breastfeeding Association's Lactation Resource Centre provides you with a range of excellent products including the peer review journal Breastfeeding Review [6], while in joining the International Lactation Consultant Association you will receive the Journal of Human Lactation as well as the ILCA eGlobe newsletter [1]. In addition, a new open access international journal, the International Breastfeeding Journal is also available [16].

Since 2002, I have been disseminating information (via email) about relevant breastfeeding issues to colleagues around the world, such as the new WHO breastfed infant growth charts, the Australian Breastfeeding record, milk banking in Australia, and the safe management of breastmilk. Emails are short, pertinent and usually contain a link to a website for further information. If you would like to join this list, please send an email to me at ellen.mcintyre@flinders.edu.au and I will add you to the list. There is no cost involved. I look forward to hearing from you soon.

## Competing interests

The author declares that she has no competing interests.

## References

[B1] International Lactation Consultants Association. http://www.ilca.org.

[B2] International Board of Lactation Consultants Examiners. http://www.iblce.org.

[B3] Lactnet. http://peach.ease.lsoft.com/archives/lactnet.html.

[B4] PubMed. http://www.pubmedcentral.nih.gov.

[B5] Cochrane Library. http://www.cochrane.org/.

[B6] Australian Breastfeeding Association. http://www.breastfeeding.asn.au.

[B7] Breastfeeding Friendly Hospital Initiative, Australia. http://www.bfhi.org.au.

[B8] UNICEF Baby Friendly Initiative. http://www.babyfriendly.org.uk/home.asp.

[B9] International Baby Food Action Network. http://www.ibfan.org.

[B10] LACTMED – Drugs and Lactation Database. http://toxnet.nlm.nih.gov/cgi-bin/sis/htmlgen?LACT.

[B11] United Nations International Children's Emergency Fund. http://www.unicef.org.

[B12] World Alliance of Breastfeeding Action. http://www.waba.org.my.

[B13] World Health Organisation. http://www.who.int.

[B14] La Leche League International. http://www.lalecheleague.org.

[B15] Pubcrawler. http://pubcrawler.gen.tcd.ie/www.html.

[B16] International Breastfeeding Journal. http://www.internationalbreastfeedingjournal.com.

